# Development and evaluation of TaqMan-based, one-step, real-time RT-PCR assays for pepper mild mottle virus detection for near source tracking and wastewater-based epidemiology validation

**DOI:** 10.1371/journal.pone.0278784

**Published:** 2022-12-19

**Authors:** Daire Cantillon, Adam P. Roberts

**Affiliations:** Department of Tropical Disease Biology, Liverpool School of Tropical Medicine, Liverpool, United Kingdom; University of Helsinki: Helsingin Yliopisto, FINLAND

## Abstract

Emergence of novel human pathogens pose significant challenges to human health as highlighted by the SARS-CoV-2 pandemic. Wastewater based epidemiology (WBE) has previously been employed to identify viral pathogens and outbreaks by testing samples from regional wastewater treatment plants. Near source tracking (NST) allows for more targeted WBE by analysing samples from individual buildings such as schools or even individual floors such as in multi-floor office buildings. Despite the public health advantages of WBE, few strategies exist for optimising NST sampling methodologies. Therefore, we developed a protocol to evaluate virus detection in NST sampling using Pepper Mild Mottle Virus (PMMoV) as a proxy for RNA viruses. PMMoV is the most abundant enteric human associated RNA virus and is present in peppers/pepper-containing foods. Two bespoke TaqMan RT-PCR assays were developed to detect a PMMoV genomic 5’ region and a capsid associated gene. To evaluate the protocol against field samples, pepper homogenates were flushed down an in-use toilet (Liverpool School of Tropical Medicine, UK) to spike wastewater with PMMoV on multiple days, and samples collected from two sewage access points to validate NST samplers. These wastewater samples were assessed for PMMoV based on Ct values and results compared to pepper and Tabasco derived PMMoV positive controls. Positive detection of PMMoV was comparable and consistent in ten independent samples across two NST samplers regardless of pepper homogenate spiking. We have developed two novel one step TaqMan assays that amplify both PMMoV targets in viral RNA extractions from peppers, Tabasco, and wastewater samples with cDNA synthesis through to RT-PCR results taking approximately 30 minutes. Pepper homogenate flushing was not required to detect PMMoV in our wastewater samples, however this strategy of flushing PMMoV containing materials outlined here could be valuable in assessing and validating NST in buildings with no previous or current sewage flow.

## 1. Introduction

Pandemics are large scale outbreaks, occurring at an international level, of an infectious disease that causes significant increases in mortality and morbidity. In addition to impacting on people’s health, a pandemic can also cause severe social and economic instability [1]. Globalisation, increased travel, changes in how land is used, and climate change are risk factors for the emergence of pandemics. These are ongoing global issues and as such the current SARS-CoV-2 pandemic is unlikely to be the last pandemic we will see in the coming decades [2]. Targeted approaches need to be implemented to tailor responses to current and future pandemics. Wastewater epidemiology (WBE) employs surveillance and monitoring of wastewater and sewage, and can act as an early warning system for identification of new variants of SARS-CoV-2 as well as detection of other pathogens such as polio virus [3], antibiotics and other chemical residues and antimicrobial resistance [4].

The technology and infrastructure required to conduct national scale WBE sampling however is relatively novel but shows promise and has gained increasing attention during the current pandemic [5, 6]. Wastewater samplers and WBE sampling regimens require field evaluations, iterative optimisation, and innovation to be as efficiently implemented in multiple settings throughout the world. Current WBE samples are typically sourced from wastewater treatment plants or sewage access points where a single timepoint sample, known as a grab sample, is taken for analysis [7]. While this single sample will be representative of a large geographical area or population and acts as a snapshot of pathogens present at that specific timepoint, this methodology is not without issues. These grab samples from wastewater treatment plants do not enable resolution on smaller catchment areas such as neighbourhoods, individual buildings or specific areas within large buildings such as high-rise offices or heavily populated urban areas in low-middle income countries. There can be high variation in pathogen abundance between households and sampling at wastewater treatment plants does not allow this variation to be captured.

Near source tracking (NST) has been developed to determine WBE at a much more focused scale. This methodology involves using samplers to collect samples over a defined period of time, known as composite samples, from one specific building, such as a school, hospital, apartment block or a prison [5]. The rationale of this strategy is that NST can be employed at individual building level, allowing for specific monitoring of outbreaks. This can lead to early detection of pathogens such as SARS-CoV-2 and targeted interventions, such as testing or quarantine, can be deployed to prevent further spread at a community or hospital level.

One specific challenge with NST is how to validate wastewater sampling technologies. Frequently, wastewater samplers will be validated by installation in a sewer access point of a building and composite samples regularly collected [8, 9]. Wastewater contains viable pathogens, including SARS-CoV-2 that may require the use of a Biosafety Level 3 containment laboratory which may not be readily available in many labs that are developing NST wastewater samplers. As field evaluations are key to developing NST methodologies we have selected a non-pathogenic virus as our target organism that can be used to evaluate NST without the need of a BSL3 laboratory.

Our project aim was to show that samplers we were tasked with testing could recover detectable RNA virus from wastewater samples. The *Pepper Mild Mottle Virus* (PMMoV) belongs to the genus *Tobamovirus* and was first isolated in 1984 from peppers [10]. It is a non-enveloped positive sense single stranded RNA virus that is a significant plant pathogen, causing mottles on the flesh of peppers and related crops [11]. We selected PMMoV as our target virus as it has previously been used a marker of human faecal contamination and as a water quality indicator so was likely to be successfully recovered [12–14]. These studies identified PMMoV by one TaqMan assay; here we report two additional novel TaqMan assays to detect different regions of the PMMoV genome. Although this agricultural pathogen has been adapted to assess water quality, there has been no studies applying PMMoV to NST development. The objective of this study was to develop rapid one-step TaqMan RT-PCR assays and evaluate these against wastewater samples with and without pepper homogenates spiked in via flushing in wastewater. PMMoV presence in wastewater is dependent on diet, so pepper homogenate was added to ensure PMMoV detection [14].

## 2. Materials and methods

### 2.1 Sample collection

Two 24h composite sampling devices (provided by Bio Data Networks Ltd) were installed at Liverpool School of Tropical Medicine, Liverpool, UK in March 2021. Both samplers were installed by Bio Data Networks Ltd in sequence at two outdoors sewage access points. Toilet flushes drained into this sewer system, passing both samplers. The first sampler (sampler 1) accumulated solid matter in a collecting bed. After 24h, a manual draw system was used to collect 50 mL of liquid containing solid matter from the collecting bed. The second sampler (sampler 2) sampled ~50 mL of each flush by activating a sampling trigger. Sampler 2 pooled these samples in to a 1L vessel and this sample pot was harvested after 24h. Sampler 2 composite 1 L sample had four 50 mL volumes transferred to four 50 mL Falcons. Sampler 1 and 2 samples were centrifuged at 2,800 g at 4°C for 30 minutes. Supernatants were stored at -80°C.

### 2.2 Processing of peppers and Tabasco

Bell peppers were purchased from a market supplier in Liverpool, UK, in May 2021. Green, yellow and red peppers were selected, with preference given to peppers showing mottling as this suggests presence of PMMoV [15]. Tabasco sauce was purchased on the same day from a supermarket (Tesco, Liverpool, UK).

Bell pepper stems were removed and cut into sections approx. 4cm x 4cm. The equivalent of three peppers were transferred to a blender and 100 mL de-ionised water added. These were blended at full speed for 1 minute to produce a fine homogenate of ~500 mL in volume. This homogenate was flushed over several flushes into a toilet draining directly into the sewer pipes containing the wastewater samplers. Pepper homogenate-free wastewater samples were collected on days prior to flushing pepper homogenates to prevent carryover between samples.

Pepper homogenate 40 mL aliquots were centrifuged at 2,800 g at 4°C for 30 minutes and the supernatants stored at -80°C. To process Tabasco, 50 mL volumes were centrifuged at 2,800 g at 4°C for 30 minutes and the supernatant stored at -80°C.

### 2.3 Virus enrichment

Supernatants from sewage samples, peppers and Tabasco that were stored at -80°C were thawed at ambient temperature. These samples were transferred to 250 mL polypropylene copolymer bottles, the pH of each sample was adjusted to 7.0–7.6 with 1M NaOH or 1M HCl and 12.5 mL of a 40% w/v PEG 8000, 8% w/v NaCl solution was added to each sample. These samples were placed in an orbital shaker at 200 RPM for 15 minutes to mix well then put on a vertical roller at 25 RPM, 4°C for 16h. Polypropylene copolymer sample pots were centrifuged at 10,000 g for 30 minutes at 4°C in an ultracentrifuge with the brake setting on low. Supernatant was removed to discard, and sample pots centrifuged at 10,000 g for 5 minutes at 4°C. Residual supernatant was pipetted to discard, and pellets re-suspended in 1 mL Trizol, transferred to a 1.5 mL RNase free Eppendorf tube and stored at -80°C.

### 2.4 RNA extraction

Trizol samples were thawed on ice and 200 μL molecular grade chloroform was added and mixed well by vortexing followed by a five-minute incubation at ambient temperature. Samples were centrifuged for 15 minutes at 12,000 g for 4°C. The colourless aqueous phase of each sample was transferred to a new 1.5 mL Eppendorf tube and one volume of molecular grade 100% ethanol added and mixed by vortexing. RNA extraction was performed using a Macherey Nagel NucleoSpin RNA extraction kit following manufacturers’ instructions. Eluted RNA quantity was immediately determined using the RNA quantification kit Qubit HS assay (ThermoFisher Scientific) and quality assessed with a NanoDrop spectrophotometer. RNA samples were subsequently stored at -80°C.

### 2.5 Primer and probe design

The genome sequence of PMMoV NC_003630 was downloaded from the National Center for Biotechnology Information GenBank database and custom gene expression TaqMan assays were designed using ThermoFisher custom TaqMan Assay Design Tool [16]. Primers and probes were designed for two PMMoV genes; a capsid gene (‘CAPS’) and a gene located at the 5’ end of the genome (’END’). These are detailed in Table 1.

**Table 1:** TaqMan assay sequences used for Pepper Mild Mottle Virus (PMMoV) detection.

Primer Name	Primer function	Assay ID	Catalog Number	Primer Sequence	Gene target
PMMOV_CAPS_F	Forward primer	APH6FF3	4331348	GAAGTTGAAAATCCGCAAAATCCTACA	CAPS
PMMOV_CAPS_R	Reverse primer	APH6FF3	4331348	CCACCGTCGCATCGTCTAC	CAPS
PMMOV_CAPS_M	TaqMan MGB probe	APH6FF3	4331348	FAM–ACGCTGTCGCTTTGC–MGB–NFQ	CAPS
PMMOV_END_F	Forward primer	APGZKV6	4331348	TTCGCACTGCACGGATAAAGTAT	END
PMMOV_END_R	Reverse primer	APGZKV6	4331348	GCCCCAAATTCATCTGCTGGAA	END
PMMOV_END_M	TaqMan MGB probe	APGZKV6	4331348	FAM–ACGCTGTCGCTTTGC–MGB–NFQ	END

FAM: 6–Carboxyfluorescein; MGB: Minor groove binder; NFQ: Non–fluorescent quencher

### 2.6 PMMoV specific RT-PCR

Absence/presence RT-PCR was performed using TaqMan Fast Virus One-Step Real Time RT-PCR Master mix (ThermoFisher Scientific). Each 12.5 μL reaction contained 1X TaqMan Fast Virus mastermix, 1X TaqMan gene expression assay and 5 μL RNA sample. Amplification reactions were performed in 96 well PCR grade microtiter plates using a QuantStudio 5 qRT-PCR machine (ThermoFisher). RT-PCR conditions were as follows: reverse transcription of 50°C for five minutes; RT inactivation/initial denaturation of 95°C for 20 seconds; denaturation of 95°C for three seconds and annealing/extension of 60°C for 30 seconds. Denaturation and annealing/extension were carried out for 40 cycles.

### 2.7 Data analysis

RT-PCR data were analysed using QuantStudio Design and Analysis Software v1.5.1 to generate amplification plots and Ct values. GraphPad v9 was used to plot column graphs.

## 3. Results

### 3.1 PMMoV is detectable in Tabasco and peppers

**Fig 1:**
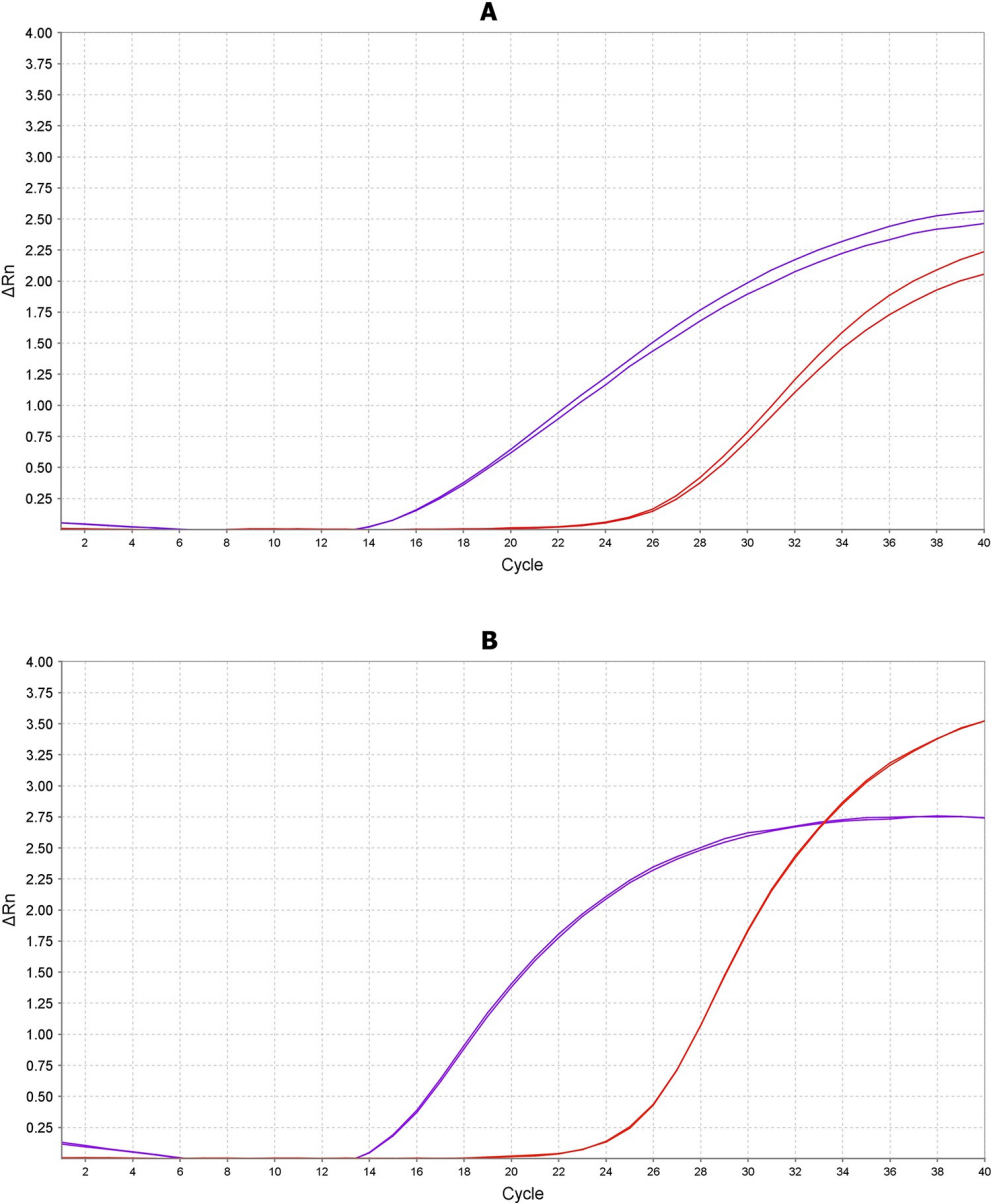
Pepper Mild Mottle Virus (PMMoV) can be detected in peppers and Tabasco. Two PMMoV genes, CAPS and END, were amplified in pepper and tabasco RNA extractions via TaqMan RT–PCR. CAPS (A); END (B) gene amplification plot showing pepper (red) and tabasco (purple). These plots reveal that amplification occurs at lower Ct thresholds in tabasco compared to pepper, suggesting that PMMoV is present at higher concentration in tabasco. Two technical replicates are plotted per condition.

We could detect PMMoV by both novel TaqMan assays. PMMoV was confirmed in the peppers selected with mottles. However, tabasco showed amplification at lower Ct values (CAPS gene: 16.479 Tabasco, 26.449 peppers; END gene: 15.062 Tabasco, 24.636 peppers) suggesting that Tabasco is a superior source of PMMoV compared to peppers with mottles, Fig 1.

### 3.2 PMMoV is detectable in wastewater

Pepper homogenates were flushed on three separate days to increase pepper (and thus PMMoV) presence in wastewater samples. No pepper homogenate was flushed during the first day of sampling for comparison. Pepper homogenate free samples were collected first to prevent carry over of pepper homogenate into this sample. These samples were taken from both wastewater samplers with supernatants stored at -80°C until processed for viral RNA extraction and RT-PCR. As four sampler 1 sample volumes were 50 mL each, two samples with no added pepper homogenate were pooled together, and two samples that had pepper homogenate spiked were also pooled together. Sampler 2 samples (200 mL each) were treated as four independent samples. These were subsequently processed for virus enrichment and RNA extractions.

**Fig 2:**
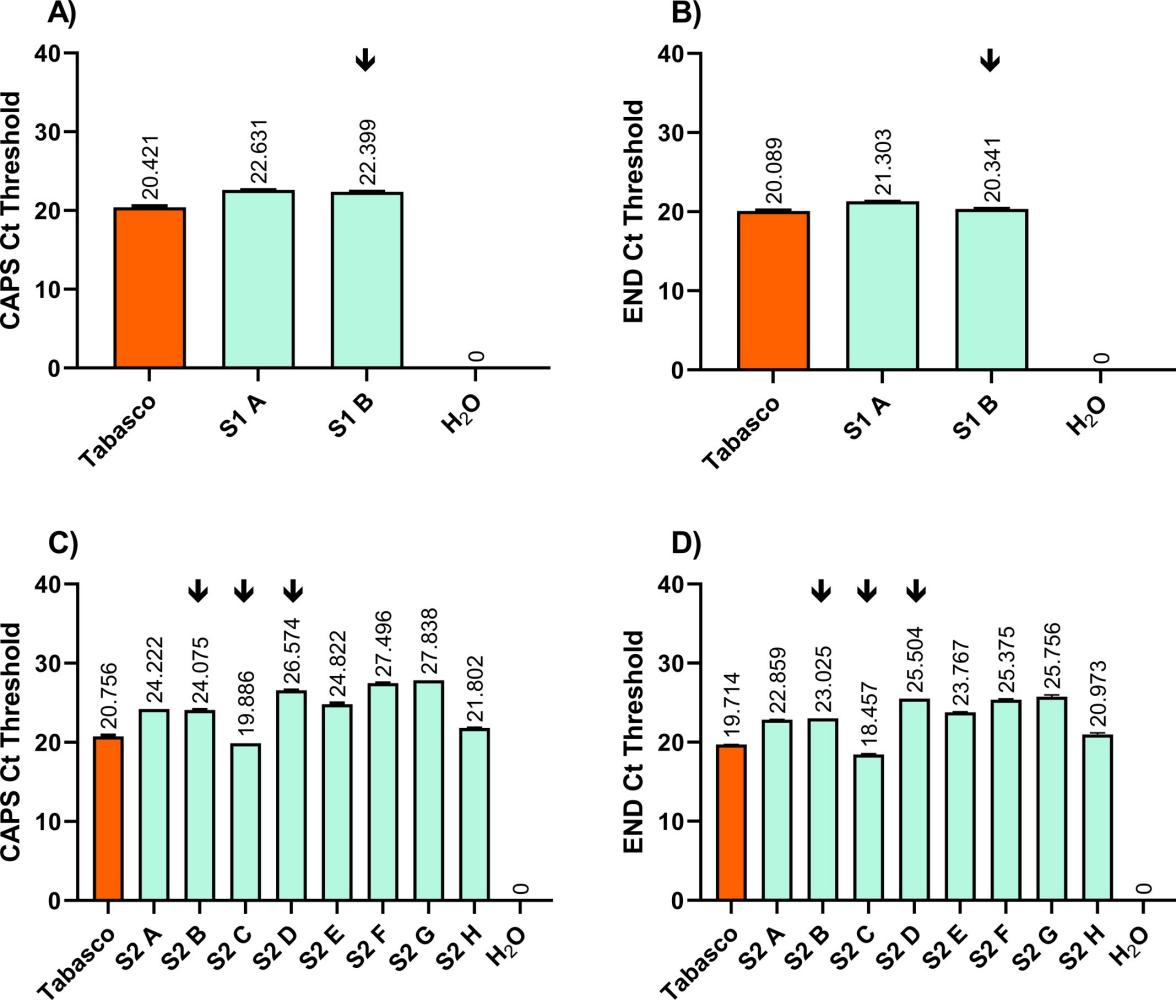
PMMoV is identified in wastewater samples. Two PMMoV genes, CAPS and END, were amplified in wastewater RNA extractions via TaqMan RT–PCR. Sampler 1 samples show A) CAPS, and B) END gene amplification, with Tabasco as a positive control. S1 A: sampler 1 sample with no pepper homogenate flushed in. S1 B: sampler 1 sample, with pepper homogenate flushed in. Sampler #2 samples also show C) CAPS, and D) END gene amplification. Tabasco was used as a positive control. S2 A–S2 H: Sampler 2 samples A–H. S2 A, S2 E–H had no pepper homogenate flushed in; S2 B–D had pepper homogenate flushed in. H_2_O is where water was used as input for RT–PCR as negative controls. Two technical replicates are plotted per column with ± standard deviation as error bars. Arrow icon indicates samples with pepper homogenate flushed in. Mean Ct values are shown over columns.

PMMoV amplification was observed for all samples tested, showing all wastewater samples collected from both sampler devices contained detectable PMMoV. Flushing in pepper homogenate to spike wastewater with additional PMMoV did not appear to alter PMMoV presence/absence RT-PCR results in wastewater samples based on Ct values, Fig 2.

## 4. Discussion

Wastewater surveillance for SARS-CoV-2 has been in place in England since June 2020, conducted by a multidisciplinary team involving governmental and UK Water utilities [7]. The sampling coverage is approximately 70% of the population of England, with sampling sites primarily through sewage treatment works and access points throughout a sewer catchment although 55 NST sites in single buildings are also in use. The authors discuss how single time point grab samples from these sites often yield poor sensitivity for pathogen detection. Composite samplers that operate continuously can collect wastewater over a longer period of time and thus increase the likelihood of detecting pathogens of interest. In this study, samples were collected by continuous sampling and PMMoV was detected in all samples tested. Continuous sampling technologies are at the cutting edge of wastewater epidemiology and our validation strategy can be applied to these new technologies. Studies [17–19] published to date using TaqMan assays to identify PMMoV have based their TaqMan primer/probe sequences on two studies [13, 14]. Haramoto et al. modified their forward primer from the Zhang et al. study to correct for a nucleotide mismatch at the 3’ end to attain full primer-target sequence matching, highlighting that TaqMan assays can be improved upon with subsequent redesign. Thus, we designed new TaqMan assays for two reasons. First, to ensure that the most up to date PMMoV genomic sequence was used in their design. Second, the ThermoFisher TaqMan design platform continuously develop their bioinformatics databases and chemistries, reducing the probability of non-specific amplification. Taking advantage of these updated features by designing novel TaqMan assays may lead to assays with increased sensitivity, specificity and precision.

We initially used bell peppers as a source of PMMoV as this fruit has commonly been shown to contain PMMoV [20]. The presence of PMMoV may vary on the geographical source of peppers, but we selected peppers that showed evidence of mottling, associated with PMMoV infection. It has been reported that Tabasco is a rich source of PMMoV, and we found this to be a much better source of PMMoV compared to peppers based on Ct values [21].

We identified PMMoV in all wastewater samples tested (two pooled sampler 1 samples and four independent samples from sampler 2). PMMoV was identified in samples with and without flushed in pepper homogenates, suggesting PMMoV is prevalent in wastewater samples from the diet of building occupants. Pepper homogenate free wastewater was sampled first, before flushing pepper homogenate on subsequent days, to prevent carryover of PMMoV derived from peppers. Detection of PMMoV in wastewater has been reported previously [12, 22], although to the best of our knowledge this is the first use of PMMoV detection in NST applications. This methodology of flushing pepper homogenates to spike samplers with PMMoV offers real world utility in assessing NST methodologies in buildings that are not currently in use or are unoccupied, such as new builds or schools during holiday closures, or in model, developmental sewage and drainage systems.

These data demonstrate that PMMoV detection can be used to validate wastewater sampler devices for recovering human associated RNA viruses without working with pathogenic viruses such as SARS-CoV-2. The advantages to this approach is that a positive control virus can be isolated from readily available foods whereas other viruses may require cell culture to extract from; PMMoV is low risk to humans and can be worked with safely in a BSL2 facility. TaqMan chemistry was selected rather than SYBR Green-based detection as TaqMan has higher sensitivity and specificity, improved reproducibility, requires less experimental optimisation and does not require melt curve analysis [23]. While we did not multiplex our two target genes in this study, multiplexing is possible with TaqMan while this is not with SYBR Green. One step virus mastermix was selected as cDNA generation and 40 cycles of RT-PCR could be performed in one reaction vessel in just under 30 minutes.

Despite these advantages, our study is not without limitations. We have used an RNA virus as a proxy for pathogenic viruses and as such there will be variation in detection of actual viral pathogens. While Tabasco was shown to be a rich source of PMMoV and this has been demonstrated previously [21], processing food and plant materials for viral RNA extraction proved challenging. While TaqMan assays are highly sensitive and specific, each probe must be designed and synthesised for each unique sequence and there is an associated higher cost to this for the more target genes being detected.

## 5. Conclusions

PMMoV has shown versatility previously as a biomarker for water purity and fecal contamination of bodies of water. Here, we have detailed a proof-of-concept method to use PMMoV as a viral surrogate for clinically relevant RNA viruses, such as SARS-CoV-2, to validate NST wastewater samplers by using two bespoke TaqMan assays designed to detect two separate regions of the PMMoV genome. While our study was determining the presence or absence of PMMoV, we think these assays could be adaptable for future studies aimed at quantifying viral levels within a NST programme or strategy.

SARS-CoV-2 is unlikely to be the last pandemic pathogen we will encounter in our lifetimes. Developing NST infrastructure now will pay dividends in the future. In addition, NST can easily be adapted to detect or monitor other viral or bacterial pathogens of concern, including bioterrorist associated pathogens and AMR [4].

## Supporting information

S1 Data (XLSX)
